# Shape morphing Kirigami mechanical metamaterials

**DOI:** 10.1038/srep31067

**Published:** 2016-08-05

**Authors:** Robin M. Neville, Fabrizio Scarpa, Alberto Pirrera

**Affiliations:** 1 Advanced Composites Centre for Innovation and Science (ACCIS), University of Bristol, Queens Building, University Walk, BS8 1TR, Bristol, UK

## Abstract

Mechanical metamaterials exhibit unusual properties through the shape and movement of their engineered subunits. This work presents a new investigation of the Poisson’s ratios of a family of cellular metamaterials based on Kirigami design principles. Kirigami is the art of cutting and folding paper to obtain 3D shapes. This technique allows us to create cellular structures with engineered cuts and folds that produce large shape and volume changes, and with extremely directional, tuneable mechanical properties. We demonstrate how to produce these structures from flat sheets of composite materials. By a combination of analytical models and numerical simulations we show how these Kirigami cellular metamaterials can change their deformation characteristics. We also demonstrate the potential of using these classes of mechanical metamaterials for shape change applications like morphing structures.

The focus of this work is a type of shape changing metamaterial structure based on Kirigami principles that behaves in a different manner from existing cellular structures.

Mechanical metamaterials are a class of multiscale structures that exhibit unusual deformation and multiphysics characteristics due to the geometry and material distribution intrinsic to their topology. Examples of mechanical metamaterials are pentamodal structures that exhibit fluid-like behaviour^1^, but also configurations with distributed and periodic units that show negative mass^2^ and compressibility^3^ features. A particular class of mechanical metamaterials is characterised by negative Poisson’s ratio^4^, also called auxetics^5^,^6^. Auxetics exhibit enhanced mechanical properties; from indentation resistance^7^ to tailored bandgap behaviour in periodic lattices^8^, transformational optics^9^ and high-amplitude vibration alleviation^10^.

A possible way to construct mechanical metamaterials with periodic and shape changing characteristics is by using Kirigami principles. Kirigami is the ancient Japanese art of cutting and folding paper widespread in South East Asia since the 17^th^ century. Using slitting and folding operations, a 2D sheet can be turned into a 3D structure^11^. This technique can be used to create a honeycomb, as already demonstrated in a patent by H. B. Dean in 1921^12^. Saito, Nojima, and Pellegrino developed a mathematical definition of the cutting patterns^11^,^13^ which allowed the creation of Kirigami honeycombs and cellular structures with complex functional geometries. They also developed the associated manufacturing techniques applied to engineering sheet materials. Another closely related field of study is “foldcore” – a group of zigzag-shaped metamaterials derived from the Miura-ori geometry. Foldcore is also created by Engineering Origami processes, which can be considered a general subset of Kirigami, and it is capable of producing interesting Poisson’s ratio effects^14^. An attractive feature of Kirigami is that it is not limited to any material or scale. The kinematics of Kirigami show large potential for manufacturing on very small scales^15^,^16^. It is worth noting that the cellular structures studied by Eidini^17^ can be modified (using a particular set of parameters, and adding extra cell walls) to produce a geometry similar to the “open par” configuration studied in this work.

The structures developed in this work are referred to as “open honeycombs”, because they do not have the closed cell of the traditional honeycomb configuration (we follow the naming convention of foams, which are also called “open” or “closed” based on their cell geometry). Figure 1 shows the concept, compared to its closed cell counterpart. The presence of the folds in the structure gives rise to extremely variable mechanical properties and behaviour, which are dependent upon the fold angle (*α*) and the fold stiffness (*k*_fold_) of the Kirigami metamaterial. The variations in fold angle result in significant volume changes. The structure is extremely anisotropic, and can assume a cylindrical shape without any secondary curvature. In the rest of the article, we will describe the manufacturing and the mechanical behaviour of this concept of metamaterial, and demonstrate some potential applications in manufacturing shape changing morphing structures.

## Kirigami Metamaterial Design and Analysis

### Dimensions

**Figure 1:**
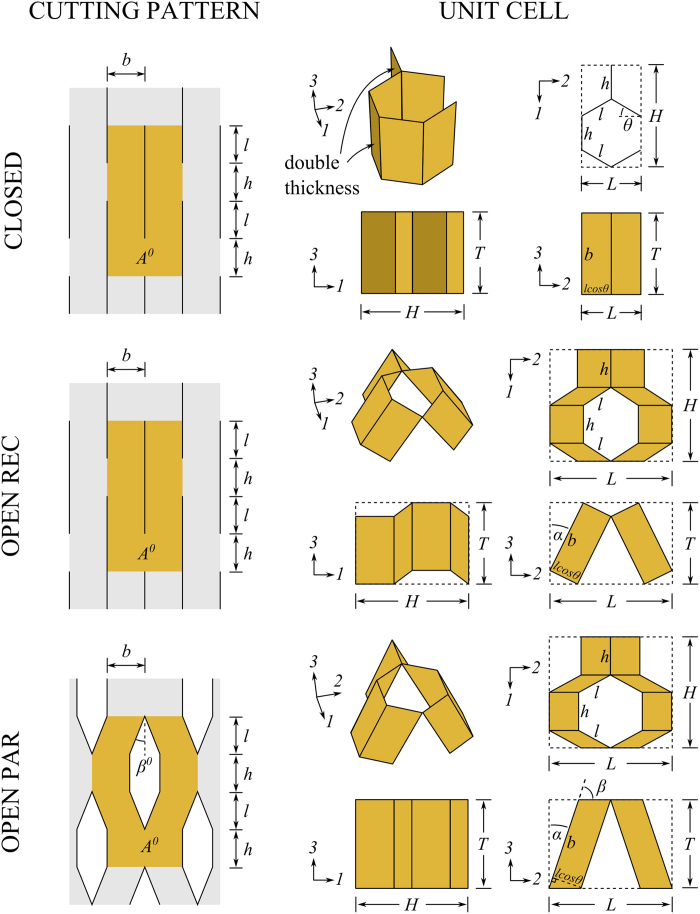
Cutting patterns and unit cells of the three honeycomb configurations. In the cutting patterns, the tan coloured patches labelled *A*^0^ represent the material used in one unit cell. The black patches represent material removed. In the unit cells, the darker walls in the closed configuration represent double thickness walls (caused by bonding together two walls).

Two variants of the Kirigami metamaterial open honeycomb were studied in this work, and compared to a traditional closed honeycomb as a baseline. The three configurations and their cutting patterns are shown in Fig. 1. To distinguish between the open configurations they shall henceforth be called “open rec” and “open par”, where “rec” and “par” are short for rectangular and parallelogram, respectively, and refer to the shape of the walls.

Because of the additional degrees of freedom associated with open honeycombs, we adopt a specific notation and terminology to define the geometry of the unit cell (see Fig. 1). For simplicity, the dimensions of the different geometric parameters used in this work are *h* = *l* = 5 mm, *b* = 10 mm, *t* = 0.25 mm, and *θ* = 30°. The shape morphing configuration is parametrised against the fold angle *α* and the fold stiffness *k*_fold_. By inspection, the overall geometry of the three different configurations can be defined by the mathematical relations shown in Table 1. The table shows two sets of equations for the open par configuration. The general case refers to any combination of *α* and *β*. When *α* + *β* = 90° the open par configuration has flat top and bottom surfaces, as seen in Fig. 1. This condition is applied to the open par configuration throughout this work. The more general form of the equations is only used to predict the theoretical Poisson’s ratios, where we assume the parts of the cell rotate with an infinitesimal variation of *α* (i.e. *α* + *β* ≠ 90°).

### Manufacturing

**Figure 2:**
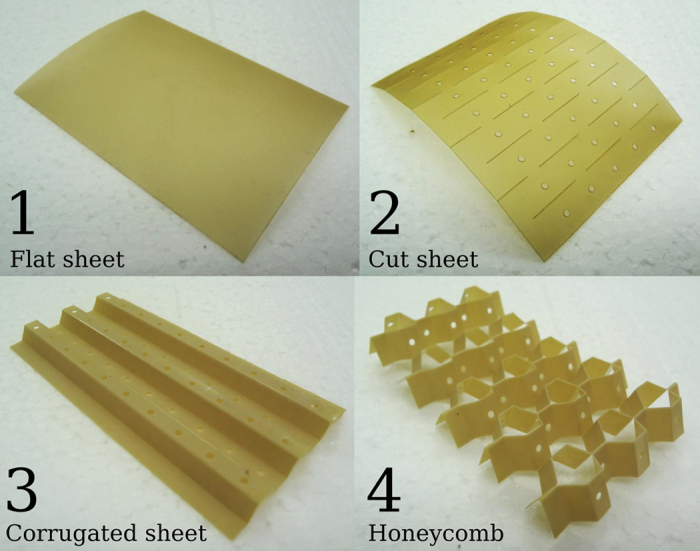
An open honeycomb at each stage during the manufacturing process.

The manufacturing process consists of three main steps: cutting, corrugating, and folding. Figure 2 shows an open honeycomb specimen at each stage of the process. The manufacturing method is versatile in terms of base engineering material, provided that the material can be cut and folded. In this work, PEEK film was used for its good formability. A pattern of slits is cut into the sheet, and it is thermoformed over hexagonal moulds such that the slits line up with the edges of the moulds. After thermoforming, the corrugated sheet is folded back on itself repeatedly such that the slits (which are now semi-hexagonal) open up into hexagonal holes, resulting in the open honeycomb geometry. Because it is relatively easy to manipulate the cutting pattern on a 2D sheet material, it is possible to include detailed features such as holes or channels into the honeycomb, which can serve a number of purposes^18^,^19^. This can also be observed in Fig. 2.

### Density

With the variation of the folding angle, the density of the honeycomb changes. By inspection, the relative density of any open configuration against the closed configuration can be calculated as:


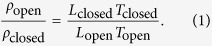


By substituting the equations for *L* and *T* for each of the configurations, the following expressions are found:


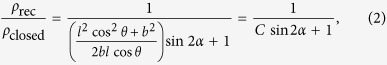



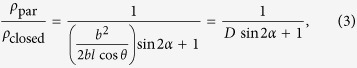


where 
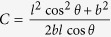
 and 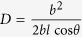. For both open configurations, the relative density reaches a minimum at *α* = 45°. By substituting the numerical values of the parameters used in this work and *α* = 45°, we obtain *ρ*_rec_/*ρ*_closed_ = 0.42 and *ρ*_par_/*ρ*_closed_ = 0.46.

### Elastic moduli

In past work^20^ we have characterised the elastic moduli of the open honeycombs compared to the closed configuration. It was found that the moduli of open honeycombs was generally lower than their closed counterparts. However, for small values of *α*, the specific moduli (e.g. *E/ρ*) of the open honeycombs were comparable to that of the closed configuration.

### Poisson’s ratios

The change of shape of the Kirigami cellular metamaterial leads to a change of the Poisson’s ratios in the equivalent continuum solid. It is possible to adapt Grima and Evans’ analysis of the Poisson’s ratio in rotating squares^21^ and apply the approach to the 3D unit cell of the Kirigami open honeycombs. When viewed in the 2, 3 plane, the cell walls appear as quadrilaterals rotating with fold angle *α* (see Fig. 1). Having already found the dimensions *L* and *T* earlier, we can use these as the *X*_2_, *X*_3_ generalised dimensions in the 3D Cartesian system. The individual Poisson’s ratio is given by the expression


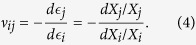


The terms *X*_*i*_ and *X*_*j*_ are functions of *α*. Using the chain rule, we obtain


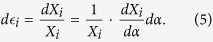



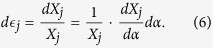


Substituting (5) and (6) into (4) we obtain the Poisson’s ratios


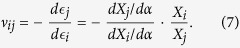


We assume that the structure deforms only by rotating about the *α*-folds, while the rest of the structure remains rigid. This approach is only valid for loading situations which produce rotations about the *α*-folds. This is true for 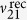, 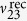, 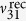, 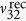, 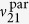, and 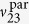. Certain loading situations prevent the strips from rotating as rigid bodies; when the open par configuration is loaded along the 3-direction for 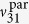 and 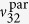 the load is spread over the top and bottom faces, preventing the strips from rotating, and when either structure is loaded along the 1-direction no moment is produced about the *α*-folds. These loading situations produce other deformations such as wall bending, and as a result the theory of rotating quadrilaterals cannot be applied. Table 2 shows the values of the Poisson’s ratios for the two configurations along the different planes.

### Finite Element Analysis

Finite element models are built to simulate the behaviour of the Kirigami metamaterial unit cell during the various phases of the shape changing behaviour. The aim of the modelling is to further compare and investigate the effect of the parameters *α* and *k*_fold_ on the equivalent mechanical properties of the honeycombs.

A model was created for each honeycomb configuration. The closed unit cell is constructed as a single entity, with the *h*-walls having double thickness (see Fig. 1). The open unit cells are built as two halves joined by hinge connectors. These connectors have a linear spring stiffness parameter which is used to represent the fold stiffness *k*_fold_. When displaying the results, *k*_fold_ is normalised by plate bending rigidity 
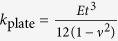
 to give an indication of fold stiffness relative to the cell wall bending stiffness.

Coupling constraints are used to link the Degrees of Freedom (DOFs) of the nodes on one edge of the unit cell with the DOFs of their counterpart on the opposite side of the unit cell. This was done to simulate the presence of neighbouring cells and thus obtain continuous honeycomb behaviour.

Three load cases are considered – one for each cartesian direction. A small compressive displacement of *δ* = 0.01 mm is introduced to the edge of the cell, and the displacements in the other two directions are recorded. These are then used to calculate the strains and the Poisson’s ratios. Because only small displacements are considered, a linear model is used.

For the closed configuration, the Poisson’s ratios can be found with one model run for each load case. For the open configurations the test matrix is larger due to the extra variables *α* and *k*_fold_. *α* is varied from 5° to 80°, and *k*_fold_ is varied from 10^−10^ to 10^10^ (i.e. from frictionless hinge to totally rigid joint). A Python script is used to build and execute the open configuration models and automatically cycle through the test matrix.

For detailed information about the FEA modelling methods, the reader is referred to the Supplementary Information.

### Experimental work

**Figure 3:**
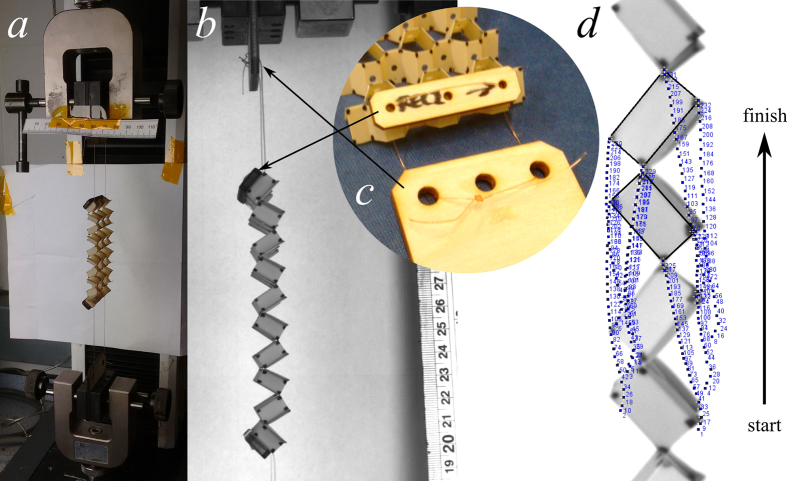
Experimental setup. (**a**) An open rec specimen in the test machine. (**b**) Still frame from the video gauge film, showing the marked corners of each cell, and the ruler used for calibration from pixels to millimetres. Inset (**c**) shows a detail view of the test fixtures. (**d**) A screen capture of the point tracking in ImageJ, showing the final frame of the video, with the previous locations of the unit cell points. The final unit cell is outlined for clarity.

To verify the predictions of the models, the Poisson’s ratios of the open honeycombs were investigated experimentally. Five honeycomb samples of the open rec configuration were manufactured and marked for motion tracking. Test fixtures were designed using cables to load the structures in tension without restraining the rotation of the cells. An Instron test machine with a 1 kN load cell was used to stretch the honeycombs in the 2-direction, and an Imetrum Video Gauge was used to film the response in the 1- and 3-directions. ImageJ software was used to measure the position of the marked points for each video frame. A Python script was used to analyse the change in XY coordinates of the points in the test. From this data it is possible to calculate the variations in cell dimensions, and from this the Poisson’s ratios were calculated. Figure 3 shows the experimental setup. A detailed decription of the experimental and analysis methods is given in the Supplementary Information.

## Results, Discussions and Applications

**Figure 4:**
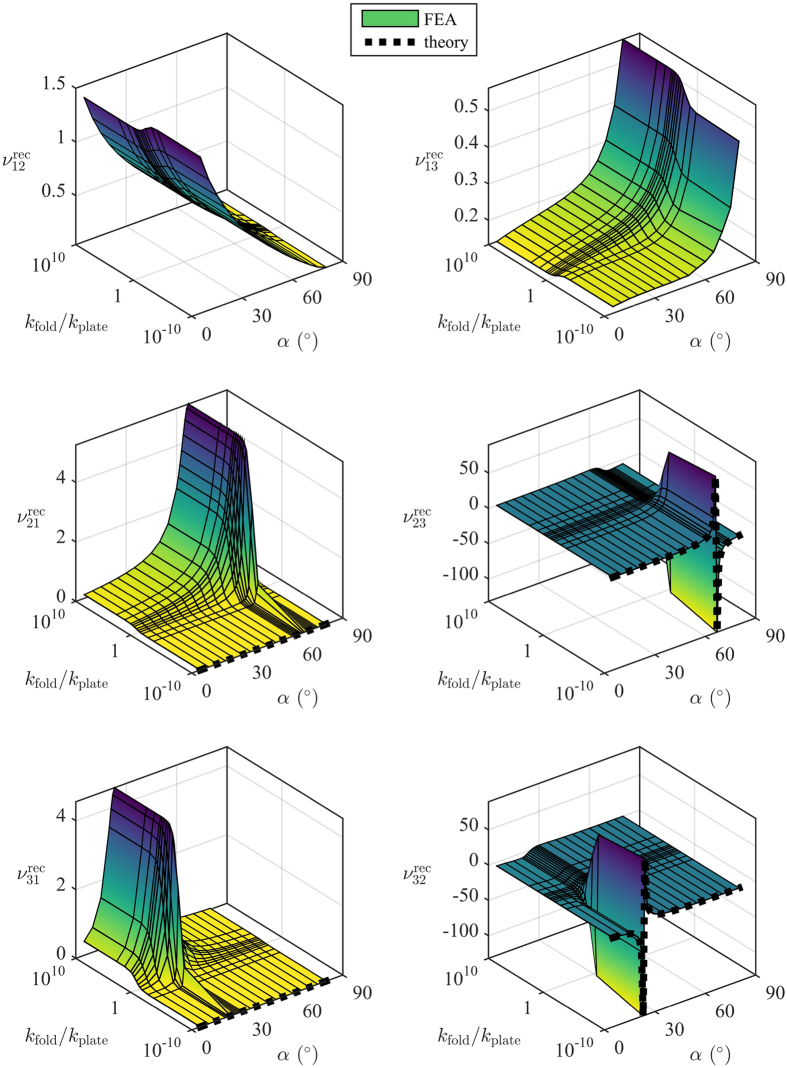
Poisson’s ratios for the open rec configuration. Note that the *k*_fold_/*k*_plate_ axis is logarithmic. The fixed honeycomb dimensions are *h* = *l* = 5 mm, *b* = 10 mm, *t* = 0.25 mm, and *θ* = 30°.

**Figure 5:**
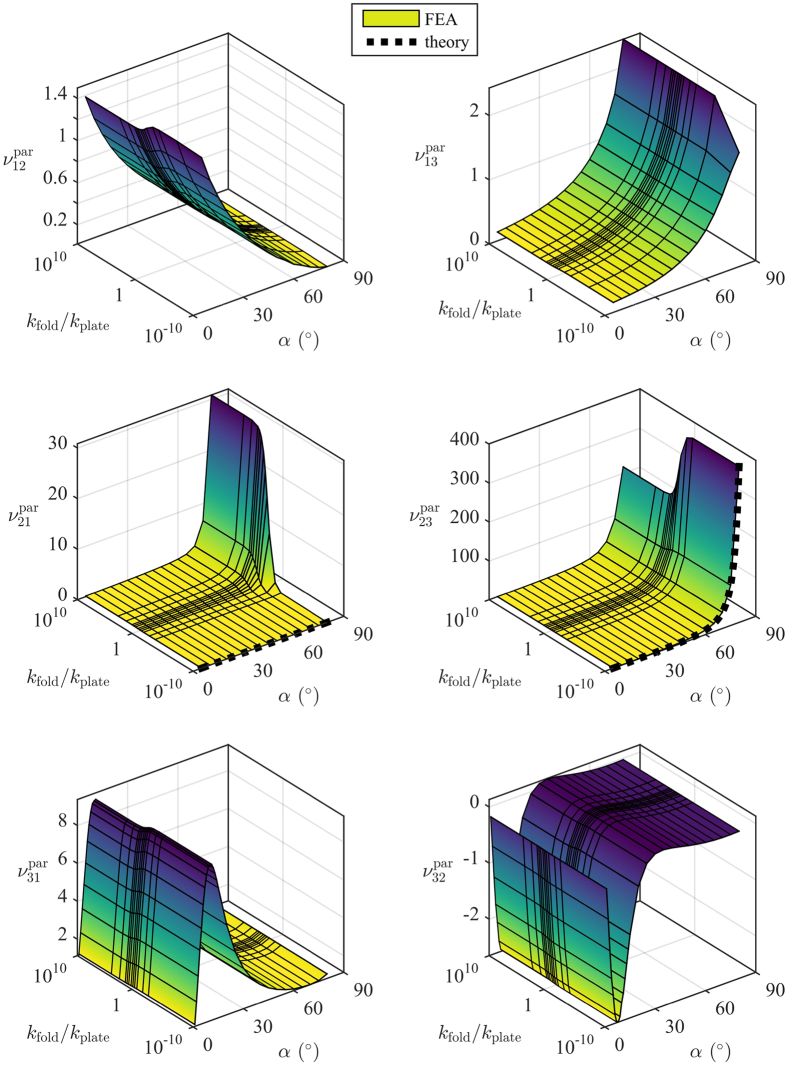
Poisson’s ratios for the open par configuration. Note that the *k*_fold_/*k*_plate_ axis is logarithmic. The fixed honeycomb dimensions are *h* = *l* = 5 mm, *b* = 10 mm, *t* = 0.25 mm, and *θ* = 30°.

**Figure 6:**
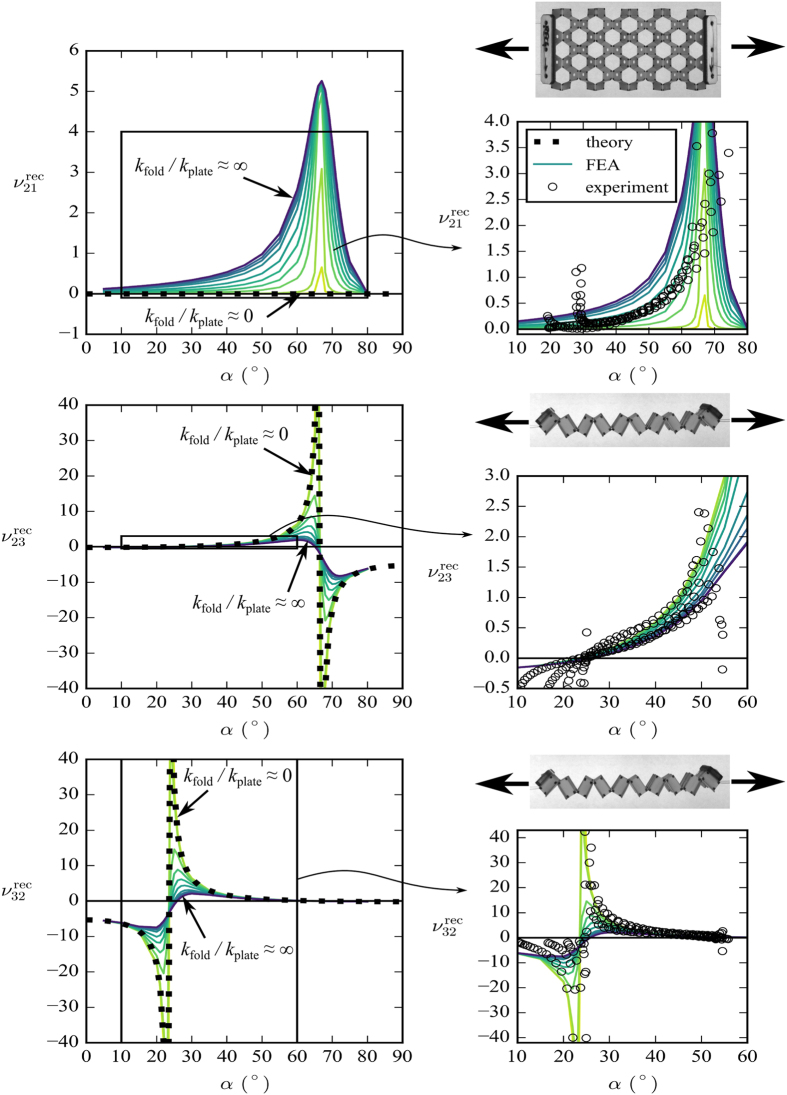
Experimental results compared to model predictions. The dotted line shows the rotating rectangles theory. The solid lines show the FEA results. The circles show the experimental results. We expect the experimental results to follow one of the FEA lines between *k*_fold_/*k*_plate_ = 0 and *k*_fold_/*k*_plate_ = ∞. The fixed honeycomb dimensions are *h* = *l* = 5 mm, *b* = 10 mm, *t* = 0.25 mm, and *θ* = 30°.

Figures 4 and 5 show the FEA and analytical Poisson’s ratio predictions for the open rec and par configurations, respectively. The surface plots show the results of the FEA, and the dashed lines show the predictions of the rotating rectangles theory (plotted at *k*_fold_/*k*_plate_ = 0 because the theory assumes no resistance to rotation). Figure 6 shows the results of the experimental work compared to the model predictions.

### Effect of *k*_*fold*_/*k*_*plate*_

From Figs 4 and 5 it can be seen that the variation of *k*_fold_/*k*_plate_ has a similar effect for all configurations and Poisson’s ratios. Ignoring for a moment the variations with *α*, and considering only the variation of *k*_fold_/*k*_plate_, all the Poisson’s ratios display three distinct behaviours:

- constant behaviour for *k*_fold_/*k*_plate_ ≪ 1
- transition behaviour for *k*_fold_/*k*_plate_ ≈ 1
- constant behaviour for *k*_fold_/*k*_plate_ ≫ 1

If *k*_fold_/*k*_plate_ ≪ 1 the folds are much less stiff than the walls, so the structure will deform primarily by deformation about the folds. For *k*_fold_/*k*_plate_ ≫ 1 the folds are much stiffer than the walls, so the structure will deform primarily by wall bending. And for *k*_fold_/*k*_plate_ ≈ 1 there will be a mixture of the two that is much more sensitive to small changes in *k*_fold_/*k*_plate_. In reality the folds will of course have nonzero stiffness. The act of folding necessarily introduces a permanent deformation into the material, which will change its stiffness relative to the pristine sheet. The question of whether the folds become more or less stiff depends on the material; for fibrous materials like paper, repeated folding often reduces the stiffness, but in other materials the folds might become stiffer via mechanisms such as strain hardening.

It is important to note that the theory of rotating rectangles matches the FEA very well for small *k*_fold_/*k*_plate_, but less well as *k*_fold_/*k*_plate_ grows larger. This means that in practice, if the folds are nearly as stiff as the walls, a finite element model will be required to capture the transition behaviour. However, if the structure can be designed such that the folds are significantly less stiff than the cell walls (by perforating or scoring the material, for example), then the theory of rotating rectangles could be used to predict the Poisson’s ratio.

### Effect of *α*

#### Cylindrical curvature

An important result is the Poisson’s ratio *ν*_21_ for both configurations. The models predict that when *k*_fold_/*k*_plate_ ≪ 1 the Poisson’s ratio *ν*_21_ is approximately zero for all values of *α*. In practice, the experimental results (Fig. 6) show that 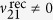, and in fact it has a small positive value, dependant on the value of *α*. This suggests that the honeycombs manufactured in this work are near the transition region with *k*_fold_/*k*_plate_ ≈ 1.

**Figure 7:**
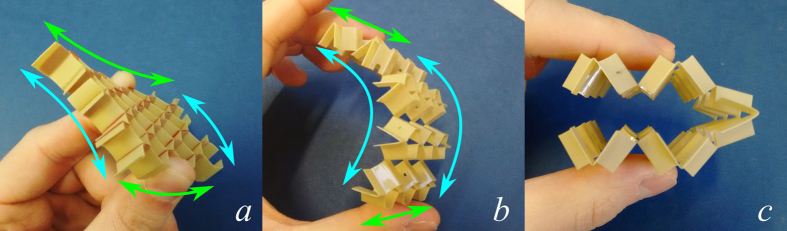
Bending behaviour. (**a**) Synclastic curvature in the closed configuration. (**b**) Cylindrical curvature in the open rec configuration. (**c**) An open rec specimen folded double.

The value of *ν*_21_ is important because a zero Poisson’s ratio here would allow the honeycomb to form a cylinder about the 1-axis with no secondary curvature about the 2-axis. Traditional honeycombs form a saddle shape when bent, which makes it very difficult to form curved components^22^. There exist several examples of zero-Poisson’s ratio (ZPR) honeycombs in the open literature^23^,^24^,^25^,^26^,^27^ but these all deform by cell wall bending, meaning that the achievable radius is limited (attempting to form small radii will crush the compression side of the honeycomb). Figure 7(b) shows that the open honeycombs presented in this work show no appreciable secondary curvature, despite having a nonzero *ν*_21_. Also, because these honeycombs deform primarily by flexing of the hinges, there is no effective limit to the radii they can form (Fig. 7(c)). This ZPR behaviour is desirable for honeycomb manufacturing and morphing/deployable structures.

#### Poisson “switch”

**Figure 8:**
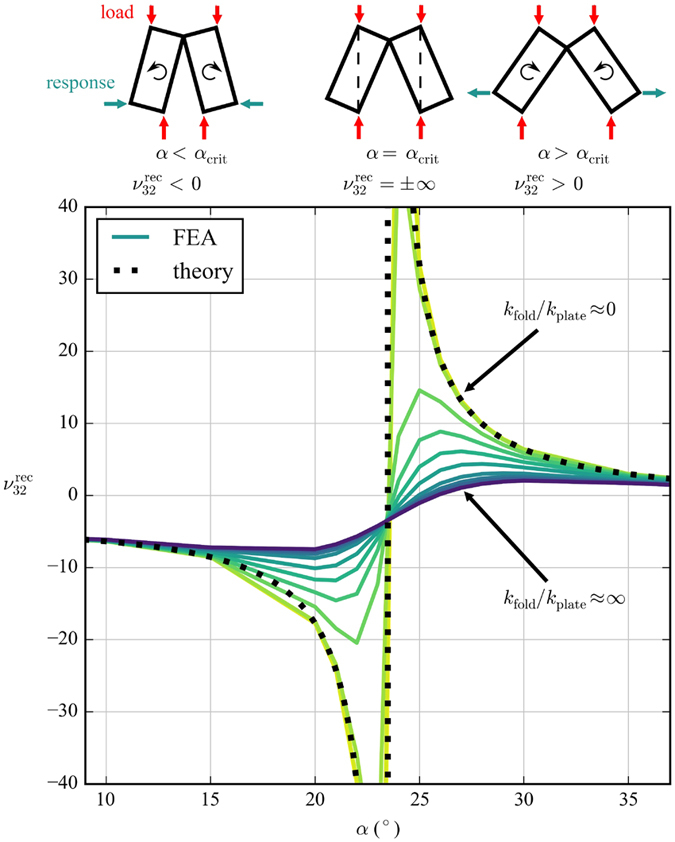
Poisson’s “switch” behaviour in the open rec configuration around the critical value of *α*. The rectangles at the top represent strips of the honeycomb (half a unit cell) viewed in the 2–3 plane, and their direction of rotation in response to a compression along the 3-direction.

Another interesting result is the Poisson’s ratios 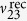 and 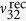. From Fig. 4 it can be seen that both Poisson’s ratios display asymptotic behaviour. This is because at a critical value of *α* the cell corners align with the loading direction, causing a change in the direction of rotation of the cells. When 
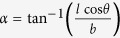
 (=23.41° for the dimensions used in this work) the corners of the cell align with the 3-direction, causing the asymptote in 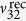. When 
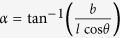
 (=66.59° for the dimensions used in this work) the corners of the cell align with the 2-direction, causing the asymptote in 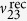. This mechanism is illustrated in Fig. 8. As *α* crosses these critical values, the Poisson’s ratio changes from negative to positive, or vice versa. *k*_fold_/*k*_plate_ affects both the sharpness of the transition and the magnitude of the Poisson’s ratio. This behaviour is interesting because it is essentially a Poisson’s ratio “switch”, and the behaviour of this switch can be controlled by both *α* and *k*_fold_/*k*_plate_. Small variations in *α* and *k*_fold_/*k*_plate_ can produce large changes in Poisson’s ratio, and can toggle the structure between positive Poisson’s ratio to auxetic. This “switch” phenomenon has been observed before in the literature, for example in the variation of the cell angle of a hexagonal honeycomb^28^. However, this behaviour is particularly interesting here because it is feasible to control *α* and *k*_fold_/*k*_plate_ *in situ*; smart material (e.g. shape memory polymer) could be used in the folds to directly control both *α* and *k*_fold_/*k*_plate_, or embedded actuators could be used to control *α* by in-plane expansion/contraction of the honeycomb. This behaviour could be very useful from a metamaterials point of view. Figure 6 shows that the experimental results also display the Poisson “switch” behaviour. For 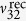 we observe a region of negative Poisson’s ratio for 10° ≤ *α* ≤ 20°, a transition region with both negative and positive Poisson’s ratio for 20° ≤ *α* ≤ 30°, and a region of positive Poisson’s ratio for 30° ≤ *α* ≤ 60°. Some experimental points in this last region appear higher than the upper bound predicted by the theory of rotating rectangles, but this is probably due to a shift in the *α*-axis. This could be caused by slight differences in dimensions between the models and manufactured specimens, or by deformations of the cell walls under load affecting the way *α* is calculated. For 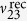 the experimental results again show good agreement with the models. Here we only observe one side of the asymptote, because the structure cannot exceed this value of *α* when loaded in tension. It can be seen that the experimental results cross the *α*-axis at the same point as the model prediction.

It is noteworthy that the open par configuration does not display any Poisson switch behaviour. This is because the geometry and loading conditions prevent the alignment of the cell corners. For 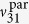 and 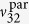 the boundary conditions were such that the cell always presented a flat top and bottom surface. The top and bottom edges were allowed to slide in the 1, 2 plane. For small values of *α*, large peaks and troughs can be seen in 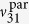 and 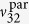 respectively, but both tend to zero as α approaches zero. At *α* = 0 the structure is like that of a closed honeycomb, and the load is perfectly aligned with the cell walls. Without any lateral perturbation, the cell walls deform axially, resulting in a zero Poisson’s ratio. For small values of *α* a small 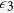 produces large 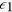 and 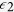, and the structure behaves in a similar way to the open rec configuration’s 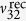 asymptotic response, but we only observe *one side* of the asymptote since *α* cannot be negative.

## Conclusions

Two metamaterial honeycomb structures have been presented, based on a Kirigami manufacturing process. The two configurations were analysed using FEA and an analytical model. The theoretical models predicted several interesting behaviours, and these were verified experimentally.

**Figure 9:**
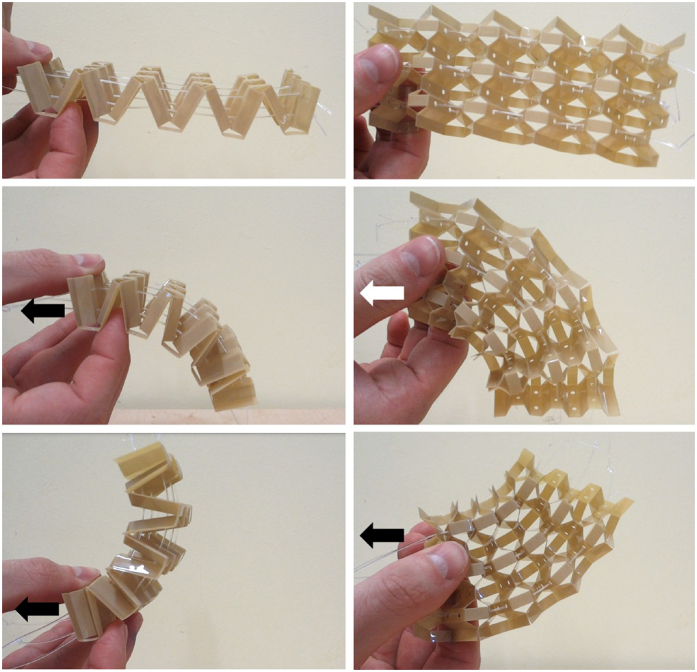
Demonstrator honeycomb changing shape in response to cable tension. The arrows indicate which cable is being pulled.

The honeycombs shown in this work provide a potential platform for unusual multifunctional and shape-changing capabilities. Circuits and sensors can be printed into the flat sheet prior to implementing the Kirigami patterning, folding and moulding. That would lead to the creation of an integrated smart active structure like the one designed for Origami robotics^29^, but with a broader range of topologies and load bearing capabilities because of the Kirigami design. It is also possible to thread cables and other inserts through holes in the cell walls. When these cables are tensioned the structure can be pulled into different deformed shapes, depending on the location of the cable within the structure (Fig. 9). Such a shape changing structure could be used for morphing airframe components^30^, or space deployable structures applications. The cables could also be used to actuate the aforementioned Poisson “switch” phenomenon.

## Methods

The Kirigami open honeycomb metamaterial has been fabricated from Victrex PEEK film. A Blackman & White Genesis 2100 ply cutter was used to apply the slitting patterns, while the corrugations were created by thermoforming the PEEK film between semi-hexagonal aluminium moulds using a modified Moore hot press at 200 °C. For the experimental testing, an Instron test machine with 1 kN load cell was used in conjunction with an Imetrum video gauge system. ImageJ was used to measure the motion of points in the videos. A detailed description of the experimental work can be found in the Supplementary Information. The analytical calculations were performed with the help of the Sympy symbolic mathematics library in the Python programming language. The Finite Element simulations have been carried out using ABAQUS v6.12 and Python. Shell elements (S4R) are used throughout the model. A convergence study has been performed to find an appropriate element size (0.25 mm). Detailed information on the FEA can be found in the Supplementary Information.

## Additional Information

**How to cite this article**: Neville, R. M. *et al*. Shape morphing Kirigami mechanical metamaterials. *Sci. Rep.* **6**, 31067; doi: 10.1038/srep31067 (2016).

## Supplementary Material

Supplementary Information

## Figures and Tables

**Table 1:** Definitions of the unit cell dimensions for the three Kirigami metamaterial honeycomb geometries.

	Closed	Open rec	Open par (general)	Open par (for *α* + *β* ≠ 90°)
*H*	2*h* + 2*l*sin *θ*	2*h* + 2*l* sin *θ*	2*h* + 2*l* sin* θ*	2*h* + 2*l* sin *θ*
*L*	2*l* cos *θ*	2*l* cos *θ* cos *α* + 2*b* sin *α*	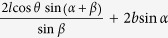	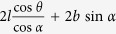
*T*	*b*	*l* cos *θ* cos *α* + *b* cos *α*	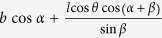	*b* cos *α*
*A*^0^	4*bh* + 4*bl*	4*bh* + 4*bl*	4*bh* + 4*bl*	4*bh* + 4*bl*

**Table 2:** Poisson’s ratios of the Kirigami metamaterial honeycomb configurations along the different planes.

	Open rec	Open par
*ν*_21_	0	0
*ν*_23_	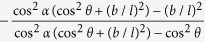	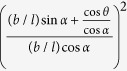
*ν*_31_	0	N/A
*ν*_32_	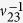	N/A
