# Supplementary material for: Shape morphing Kirigami mechanical metamaterials
Source: Sci Rep. 2016 Aug 5;6:31067. doi: 10.1038/srep31067 (PMC4974615; doi:10.1038/srep31067)
Supplement: Supplementary Information [file srep31067-s1.pdf]

Supplementary information

Manuscript title: Shape morphing Kirigami mechanical metamaterials.
Authors: R. M. Neville, F. Scarpa, A. Pirrera.

June 1, 2016

This document details the Finite Element models and the experimental methods & analysis reported in the article titled "Shape morphing Kirigami mechanical metamaterials".

1 Finite Element Analysis

An equivalent unit cell model was used, with Boundary Conditions (BCs) and constraints applied to simulate the behaviour of the actual continuous honeycomb. The unit cell model was made up of two semi-hexagonal strips, which were joined by Abaqus connectors. Figures 2-7 show the BCs and constraints used for all combinations of configuration (rec,par) and load case (1,2,3 directions).

The Abaqus "revolute" connector was used to model the folds. This connector couples the Degrees of Freedom (DOFs) of two nodes. The DOFs of the two nodes are constrained such that they behave like a cylindrical slider as shown in Figure 1. When creating the connector, a local "1" axis is defined (in this case parallel to the fold line), and the connector uses this as the "slider" axis. Relative rotations about the 2,3 axes (U_5, U_6) are restrained, and a linear torsional spring behaviour is applied to rotations about the local 1 axis (U_1), with stiffness term "D_{44}". Abaqus connector stiffness terms link the forces and moments to the displacements as per equation (1).

$$F_i = \sum_j D_{ij} U_j \tag{1}$$

In this case, D_{44} links the moment F_4 to the rotational displacement U_4, and hence D_{44} represents the fold stiffness k_{fold}. Further information can be found in the Abaqus documentation, under section Elements ≫ 31 Connector Elements ≫ 31.1 Connector elements ≫ 31.1.5 Connection-type library.

Equations were used to couple any DOFs not constrained by the revolute connector.

BCs were used to introduce translational displacements to the outer edges of the model. On one side the DOFs were set to zero, and on the opposite side the displacement δ was introduced. The BCs for each configuration and load case can be seen in Figures 2-7.

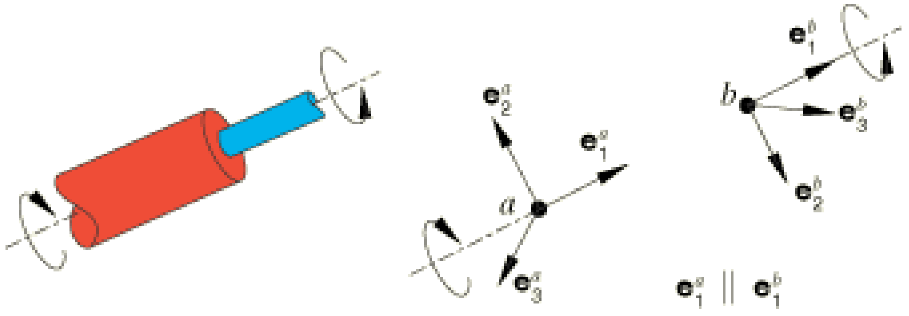

Figure 1: The Abaqus revolute connector. Image from the Abaqus user manual.

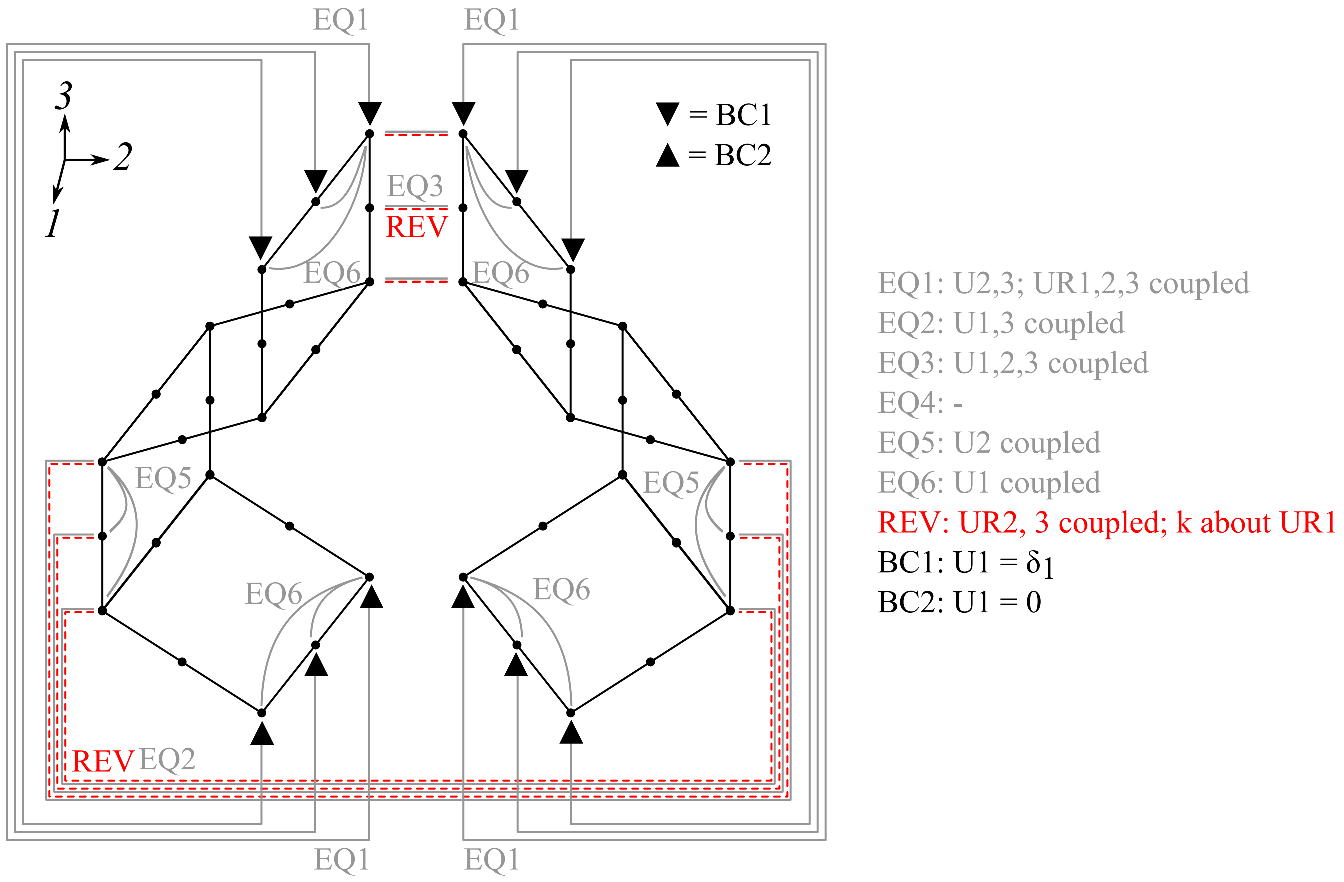

Figure 2: Configuration: rec; load case: ν_{12}, ν_{13}. “EQ” = equation; “REV” = revolute connector; “BC” = boundary condition.

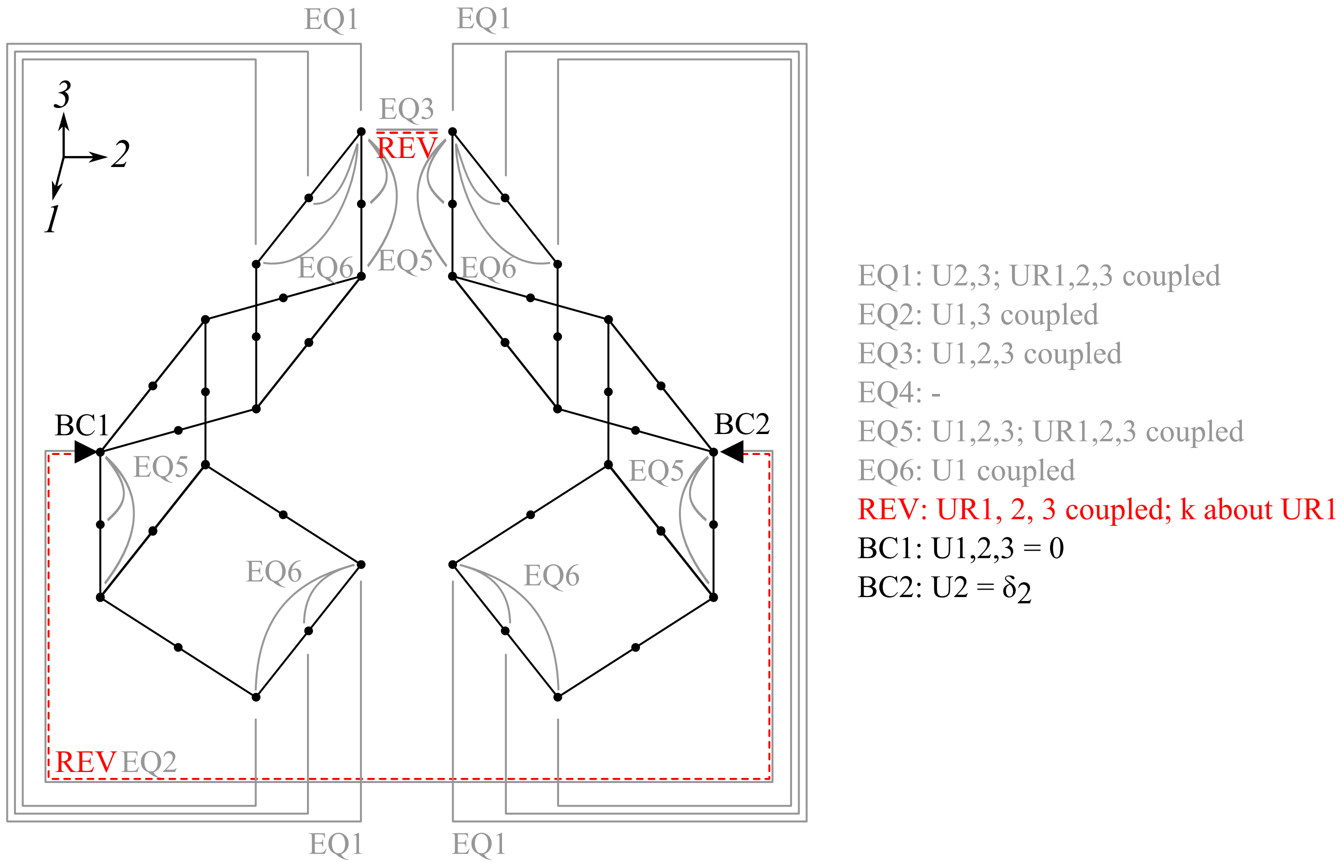

Figure 3: Configuration: rec; load case: ν_{21}, ν_{23}.

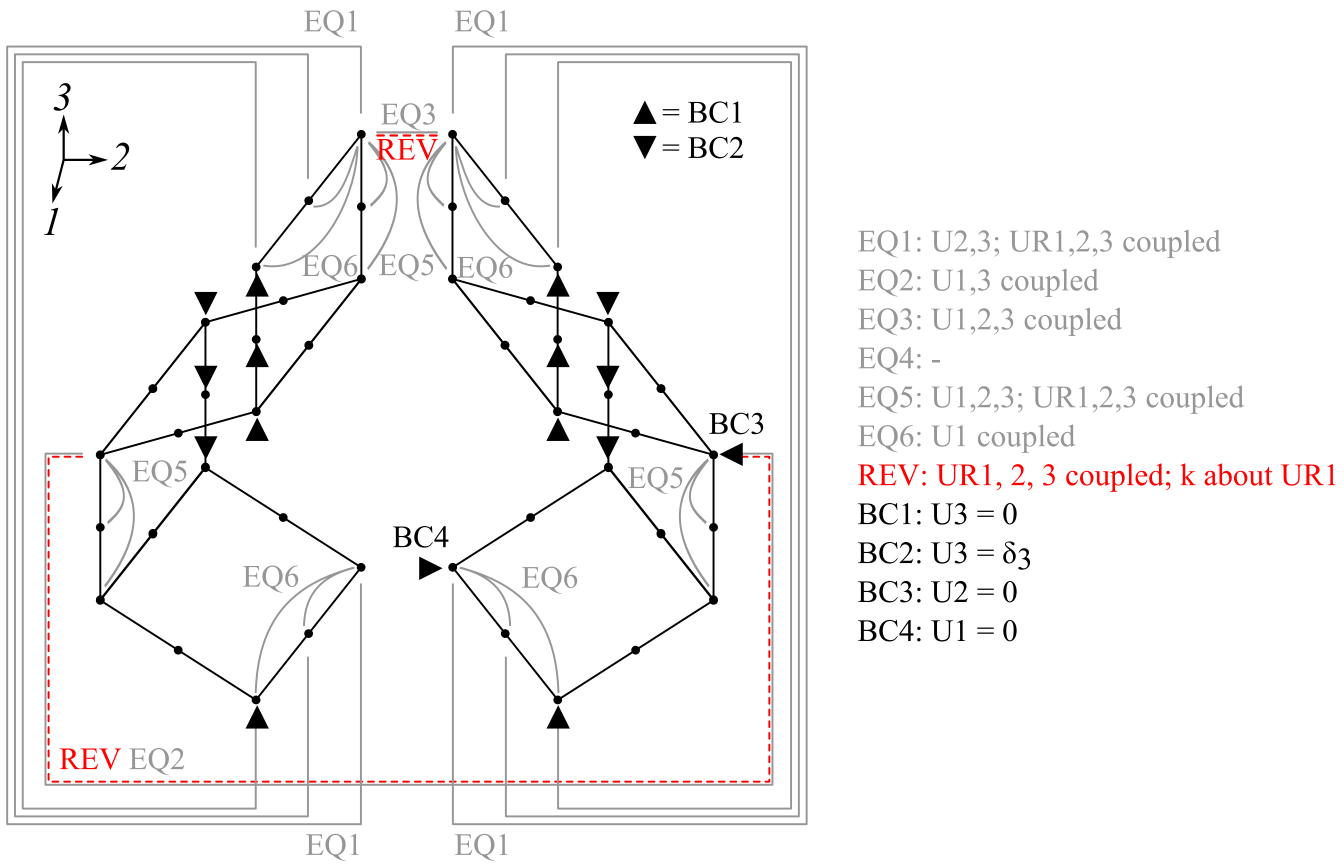

Figure 4: Configuration: rec; load case: ν_{31}, ν_{32}. "EQ" = equation; "REV" = revolute connector; "BC" = boundary condition.

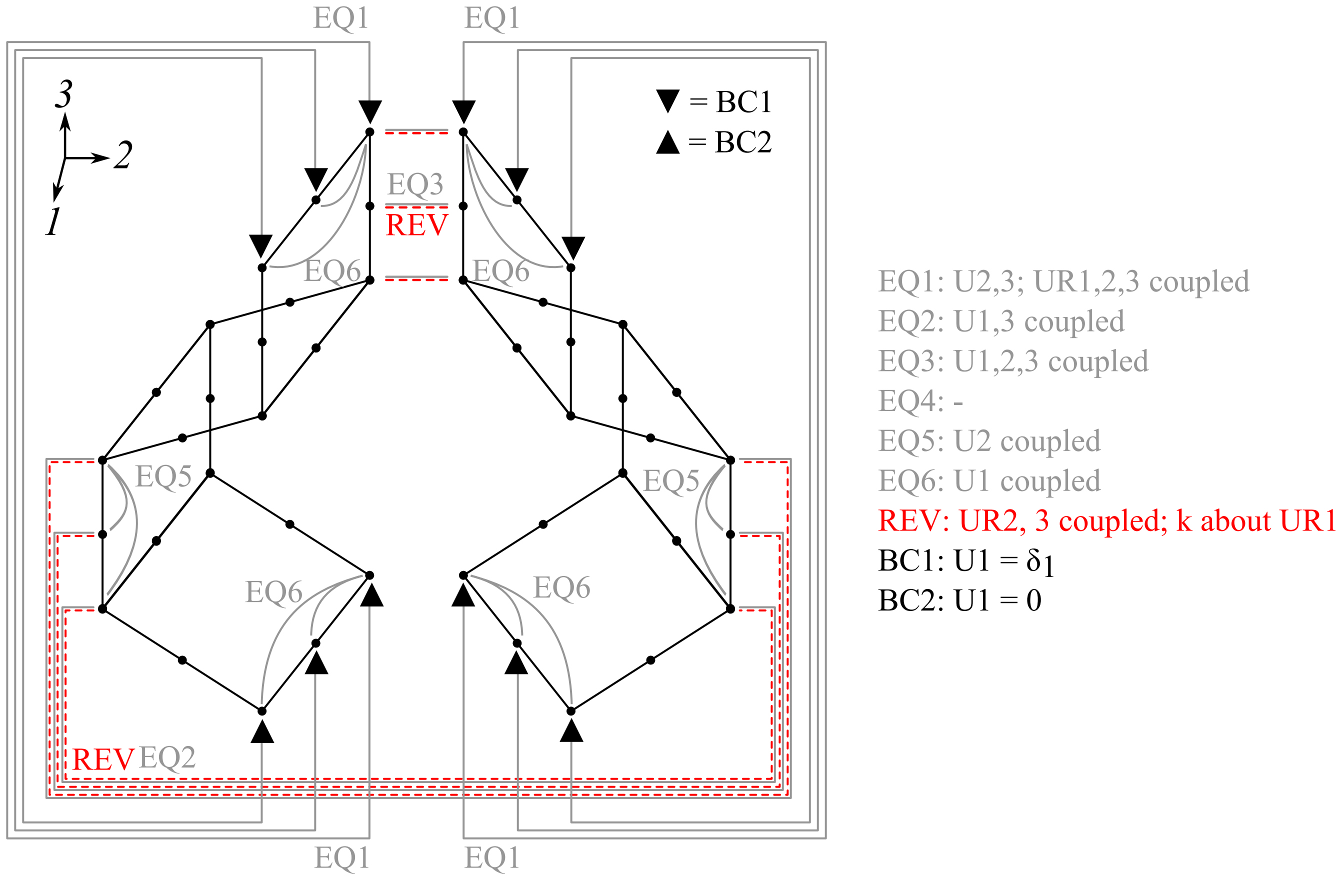

Figure 5: Configuration: par; load case: ν_{12}, ν_{13}. “EQ” = equation; “REV” = revolute connector; “BC” = boundary condition.

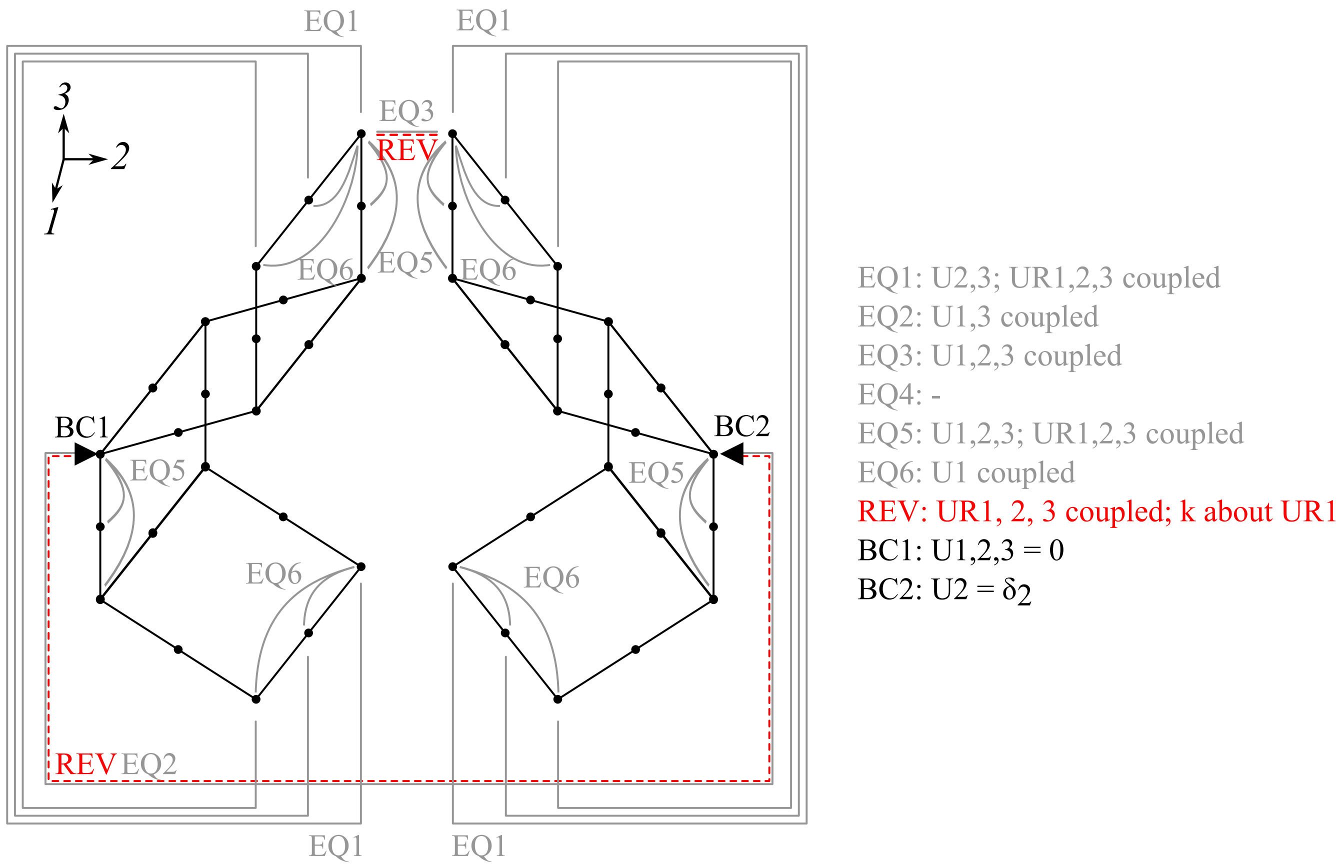

Figure 6: Configuration: par; load case: ν_{21}, ν_{23}. "EQ" = equation; "REV" = revolute connector; "BC" = boundary condition.

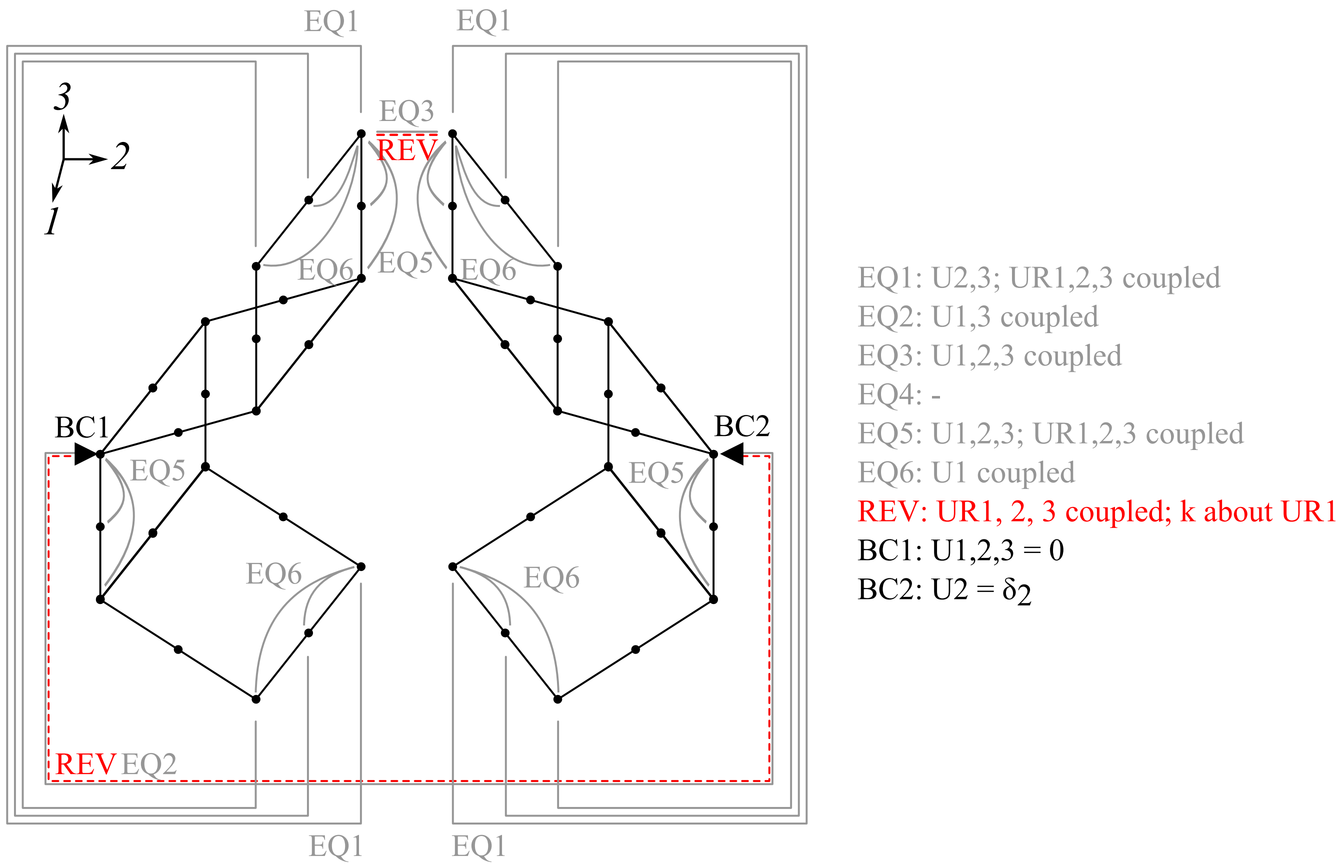

Figure 7: Configuration: par; load case: ν_{31}, ν_{32}. “EQ” = equation; “REV” = revolute connector; “BC” = boundary condition.

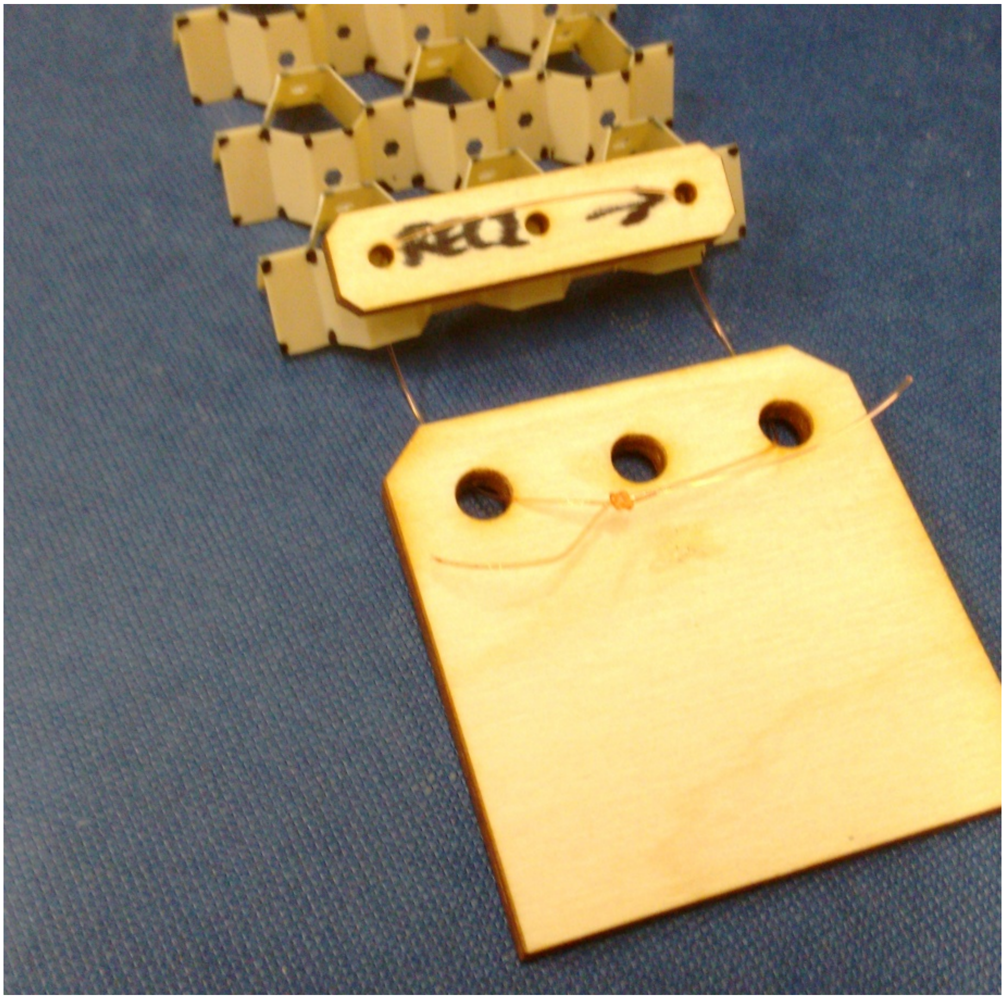

Figure 8: Self-aligning fixtures

2 Experimental work

This section describes in detail the experiments and analysis performed to verify the Poisson's ratio behaviour of open rec honeycombs.

2.1 Specimens

Five specimens of the open rec honeycomb were manufactured using Victrex PEEK 2000 series Aptiv film with 0.25 mm thickness. The cutting patterns were made using a Blackman & White Genesis 2100 ply cutter. The corrugations were formed by thermoforming the PEEK film between semi-hexagonal aluminium tooling in a modified Moore hot press at 200°C. The corrugated specimens were folded by hand to form the open honeycombs. The vertices of each specimen were marked using a permanent marker pen to serve as tracking targets for the video analysis. Holes were included in the cutting patterns to allow the specimens to be loaded using cables.

Self-tightening and self-adjusting fixtures were designed using cables. These fixtures were used to load the specimens in tension without suppressing rotation of the strips. Figure 8 shows a fixture attached to one end of a specimen. The large plate is gripped by the test machine. The smaller plate introduces the load to the specimen, and prevents the end of the specimen bunching up due to cable tension. The fixtures were laser cut from 3 mm plywood, and fishing line was used for the cables.

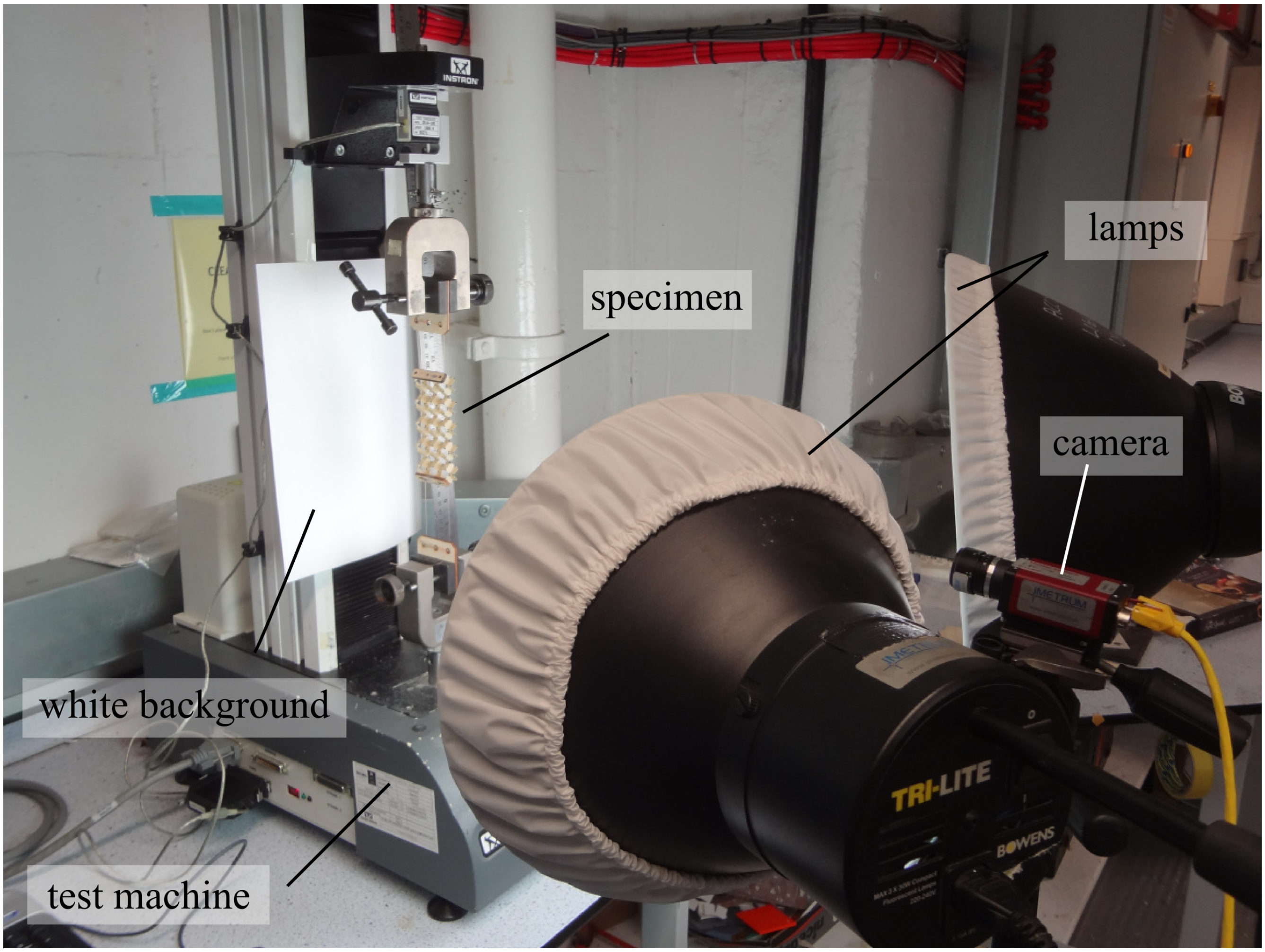

Figure 9: Experimental setup.

2.2 Experimental setup

Figure 9 shows the experimental setup used for this work. An Instron test machine with a 1 kN load cell was used to stretch the honeycombs in the 2-direction, and an Imetrum Video Gauge was used to film the response in the 1- and 3-directions (separate tests were performed for each orientation). A ruler was included in the camera shot to allow for calibration from pixels to mm.

2.3 Analysis

ImageJ software was used to measure the XY coordinates of the marked points for each video frame. A distance of 100 mm was also measured (on the ruler included in each video) to serve as a calibration from pixels to mm. Figure 10 shows the locations of the points used in the analysis. The central cell/strip was chosen to minimise any edge effects.

The lower half of Figure 10 shows how the relevant cell dimensions are calculated using the relative position of the measured points. The width and height of the cell are found from the maximum & minimum X & Y coordinates of the points. For orientation 1, α can be measured by finding the angle between the line connecting points 2 & 3, and the horizontal. For orientation 2, α is not directly visible but can be estimated by measuring the distance between points 2 & 3, which is $b \sin \alpha$ when projected onto the 12-plane. ϕ_{13} is used to identify when the critical value of α has been reached. When ϕ_{13} is zero, points 1 & 3 are aligned in the 3-direction, and this is where we expect an asymptote in ν_{32}.

A Python script was used to analyse XY data from ImageJ and perform calculations on each frame of video. Figure 11 and Figure 12 show step by step the analysis for orientations 1 & 2.

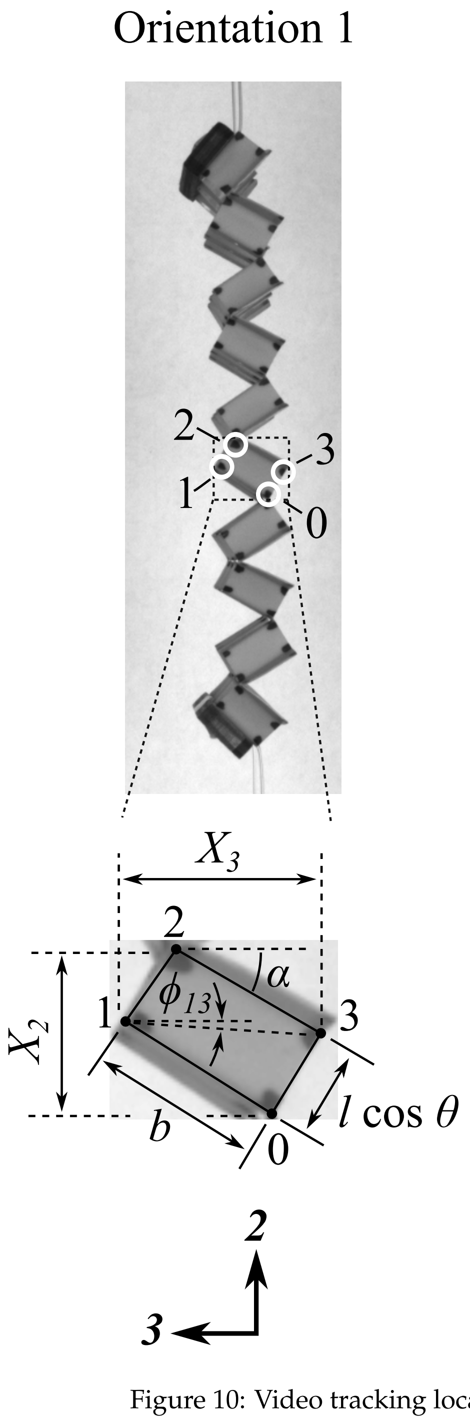

Figure 10: Video tracking locations for the two test orientations.

The raw data from ImageJ is shown in the top left plot, with the cell shapes from Figure 10 plotted on the first and last frame. This shows how the tracked cell moved during the test.

Each frame is centred to eliminate the vertical drift caused by the stretching of the specimen. The centred XY data ("x_bar" and "y_bar") are plotted in the top right plot. Having eliminated the vertical drift, the Poisson's ratio behaviour can be observed in the different bounding box shapes of the initial and final frames. In orientation 1, the rotating motion of the cell also becomes clearly visible.

Due to the resolution of the videos extracted from the video gauge system, there is some noise in the data. The noise can obscure the results if we try to calculate the Poisson's ratio over too small an interval. A third order polynomial was fitted to the data to eliminate the noise and allow the Poisson's ratio to be sampled over small intervals. The first two rows of small plots show the raw XY coordinates of the centred points compared to the curve fit. The R^2 values are all very close to 1, which indicates a good fit. The exception to this is x_bar for points 2 & 3 in orientation 2. These appear very noisy because these two points move very little in the x-direction. This is of no consequence because the x_bar of these points is not used for any calculations.

The third row of small plots shows the dimensions calculated using the curve-fitted centred coordinates – the X- and Y-widths, α, and ϕ_{13} (ϕ_{13} was only calculated for orientation 1).

The final row of plots shows the strains and Poisson's ratios calculated using the X- and Y-widths. For orientation 2, "nu_xy" (ν_{12} in specimen coordinates) was not used, because the specimen was loaded in the 2-direction, and a different wall-bending mechanism produces ν_{12} when loaded in the 1-direction. For orientation 1, both "nu_xy" and "nu_yx" (ν_{32} and ν_{23} in specimen coordinates) were used, because both are generated by rotations of the strips, which can produced by loading only in the 2-direction.

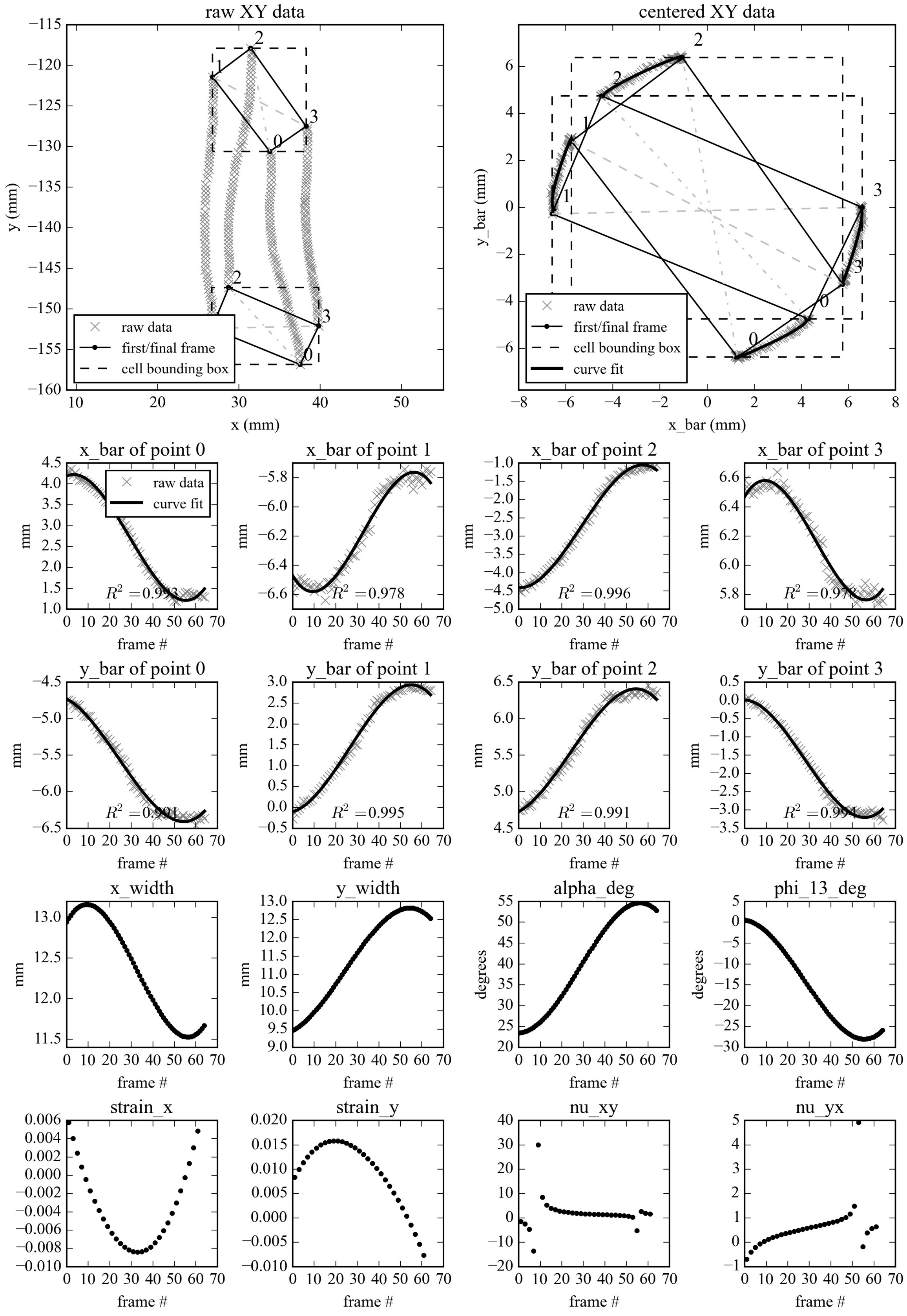

Figure 11: Analysis for one specimen in orientation 1.

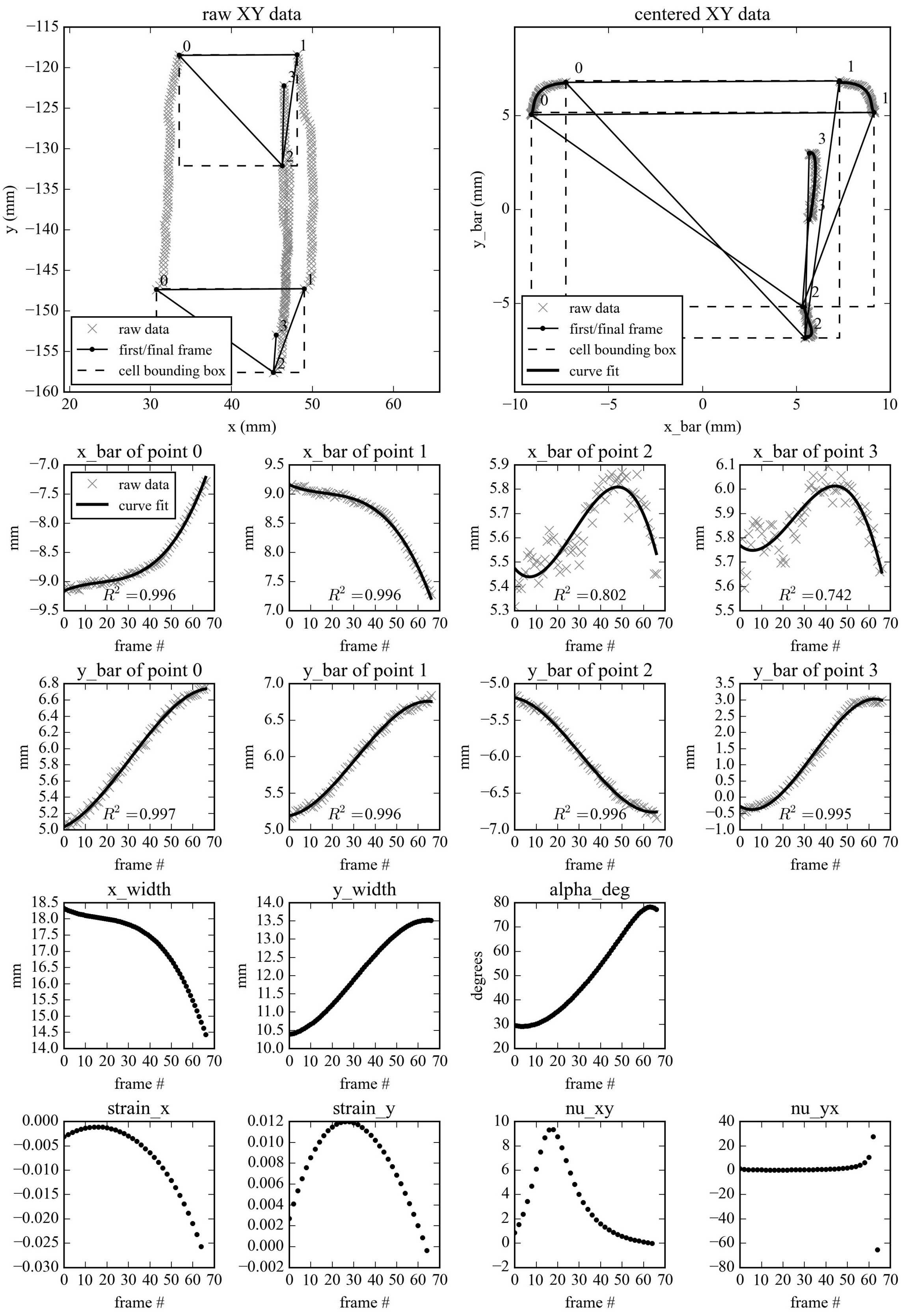

Figure 12: Analysis for one specimen in orientation 2.
